# Automatic segmentation of coronary plaques in coronary CT angiography using neural networks

**DOI:** 10.1371/journal.pone.0343887

**Published:** 2026-02-24

**Authors:** Mahdi Moosavi, Keno Bressem, Rafael Adolf, Anastasiya Valentik, Albrecht Will, Eva Hendrich, Martin Hadamitzky

**Affiliations:** Department of Cardiovascular Radiology and Nuclear Medicine, German Heart Center, TUM University Hospital, Munich, Germany; James Cook University Hospital, UNITED KINGDOM OF GREAT BRITAIN AND NORTHERN IRELAND

## Abstract

Rapid and accurate detection of coronary plaques on CCTA is critical for timely CAD diagnosis but is limited by reader workload and interobserver variability. Our objective was to evaluate the effectiveness of machine learning (ML) based on automated segmentation of coronary plaques in coronary computed tomography angiography (CCTA). We retrospectively analyzed CCTA scans from 1,642 patients (4,711 training vessels, 1,112 test vessels, plus 1,613 negative vessels) using an nnU-Net 3D full resolution architecture. Hyperparameters (batch size, learning-rate scheduler, epochs) were optimized on a 10% subset of the training dataset, and the final model was trained with 5-fold cross-validation on positive cases. Test performance was assessed at the plaque, vessel, and exam levels. Of 2,090 ground-truth plaques, the model achieved 1,772 TP for 84.8% sensitivity (95% CI 83.2–86.3%), 382 FP for 82.3% precision (95% CI 80.6–83.8), and a median Dice score of 0.88 (95% CI 0.87–0.89). At the vessel level, sensitivity was 94.7% (95% CI 93.2–95.9%) and specificity was 84.9% (95% CI 83.0–86.5%). At the examination level, sensitivity was 97.4% (95% CI 94.5–98.8%) and specificity was 79.7% (95% CI 76.5–82.6%). Detection of small, non-calcified plaques remained challenging, and false positives were often due to motion or step artifacts. Our model showed promising results in detecting coronary plaques in CCTA examinations, particularly for medium to large plaques and high negative predictive value for ruling out significant CAD, offering potential to streamline radiology workflows. Future work will focus on small-plaque detection, artifact robustness, and multi-center validation to enhance clinical utility.

## Introduction

Coronary artery disease (CAD) remains a major cause of preventable mortality, particularly among older individuals, with its incidence continuing to rise globally [1,2]. Accurate estimation of disease burden is crucial for identifying patients at high risk of major adverse cardiac events [3]. Coronary computed tomography angiography (CCTA) has emerged as a powerful noninvasive diagnostic tool for quantifying CAD [4,5], with recent guidelines emphasizing its role as the primary modality for assessing CAD in symptomatic patients [6].

Despite CCTA’s capability for volumetric CAD quantification, clinical practice continues to rely on visual assessment to determine the composition and extent of CAD [7,8]. Quantitative plaque assessment remains labor-intensive, as it requires evaluation of multiple coronary segments and plaque components by trained readers, which limits scalability in routine clinical practice [9]. This limitation highlights the need for more efficient and accessible diagnostic strategies.

The integration of deep learning (DL) into cardiovascular imaging have shown potential to improve diagnostic accuracy and patient outcomes [10,11]. ML algorithms can rapidly and accurately analyze large datasets, providing physicians with invaluable insights [12–14]. Deep learning has been successfully applied to various aspects of cardiovascular imaging, including the diagnosis of acute ischemic stroke through CT angiography data analysis [15], and the automation of complex image segmentation tasks essential for identifying vascular pathologies [16,17].

By automating plaque detection, ML based approaches may support more efficient image interpretation workflows and facilitate earlier assessment of coronary artery disease. While imaging acquisition represents a fixed cost, automated plaque detection can reduce downstream interpretation time and labor-related expenses, consistent with economic analyses reported for automated pulmonary nodule detection [18]. However, the application of deep learning models to automate coronary plaque segmentation remains underexplored. At the time of this study, no directly comparable coronary plaque segmentation models with publicly available implementations were available that could be applied to our dataset for direct head-to-head evaluation. One established approach is the use of semantic segmentation frameworks such as nnU-Net, which are specifically designed for medical image segmentation and automatically adapt to diverse biomedical imaging datasets without requiring manual parameter tuning [19]. This research aims to investigate the efficacy of a neural network-based approach in automating coronary plaque segmentation and evaluate its impact on improving the diagnostic accuracy and accessibility of CCTA. By focusing on this ML application, we seek to contribute to ongoing efforts to optimize cardiovascular diagnostic workflows, potentially reducing healthcare costs and improving patient outcomes through earlier detection and intervention of coronary artery disease.

## Materials and methods

In this study, to reduce the selection bias, we enrolled 1642 consecutive patients who underwent CCTA for suspected coronary artery disease (CAD) between 2014 and 2018 in German Heart Center of Munich, TUM University Hospital. Patients with stent implantations or coronary bypasses were excluded from analyses, as the scope of the study was to detect plaques in individuals without known CAD. All patients gave written informed consent before the investigation. The data acquisition protocol was approved by the Clinical Ethics Committee of the University Hospital rechts der Isar, Munich. All procedures were carried out in accordance with the relevant guidelines and regulations.

Post hoc analyses within the hospital do not require patient consent according to local legislation (Bayrisches Krankenhausgesetz, Art 24).

### Image acquisition

CCTA examinations were performed using a 2x192-slice dual-source SOMATOM Force CT scanner (Siemens Medical Solutions, Erlangen, Germany). Patients were placed in a supine position and received intravenous Metoprolol if heart rate exceeded 60 beats/min and Nitroglycerin if systolic blood pressure was above 100 mmHg, barring contraindications. Prospective ECG-synchronized CTA of coronary arteries was performed during inspiration at the end-diastolic phase (70% of RR interval). Tube voltage was set at 120 kV, with tube current adapted automatically based on body size using CARE Dose. After calculation of circulation time using a test bolus, Contrast media (Imeron 350, Bracco Imaging GmbH, Konstanz, Germany) was administered followed by a 50 ml saline chaser using a flow rate of 5 ml/s. If this method failed to provide diagnostic images, the patient underwent another imaging procedure using a prospective ECG-gated method to acquire multiple segments of coronary arteries during diastolic phases. This method offered more reliable image acquisition but could produce step artifacts due to multiple acquisitions. Original CCTA images were acquired and reconstructed at an in-plane resolution of 512 × 512 pixels, with the number of axial slices varying according to the scan field of view.

### Annotations and database preparation

The coronary artery tree was initially segmented using commercially available software (Syngo.via, Siemens Healthineers, Erlangen, Germany) and manually corrected by an experienced radiologist. Vessel regions containing non-calcified and partially calcified plaques were manually annotated. Calcified plaques were automatically annotated using a simple HU-based thresholding algorithm along vessel centerlines; however, this approach captured only the high-density calcified component and did not delineate the full plaque extent [10]. Individual coronary arteries were then extracted from the coronary tree and used as independent 3D inputs for model training and evaluation.

**Fig 1:**
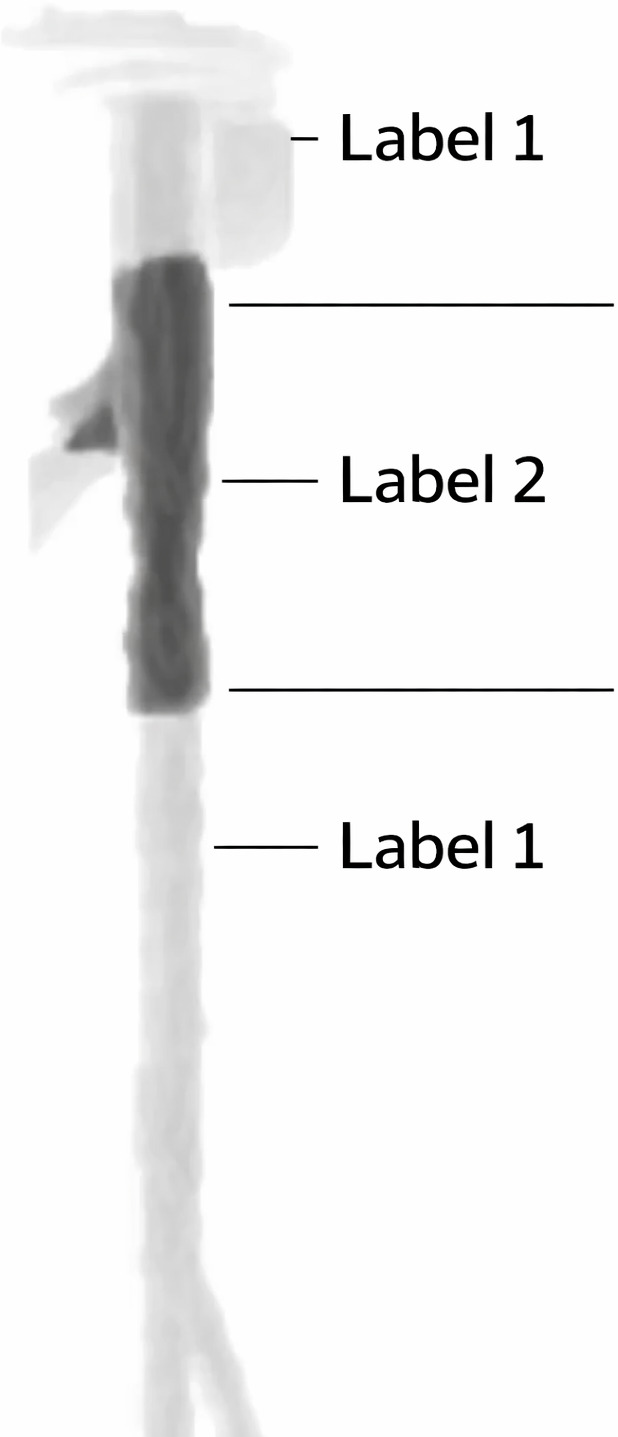
Annotation example. Non-pathological segments labeled as 1 and the plaque labeled as 2.

**Fig 2:**
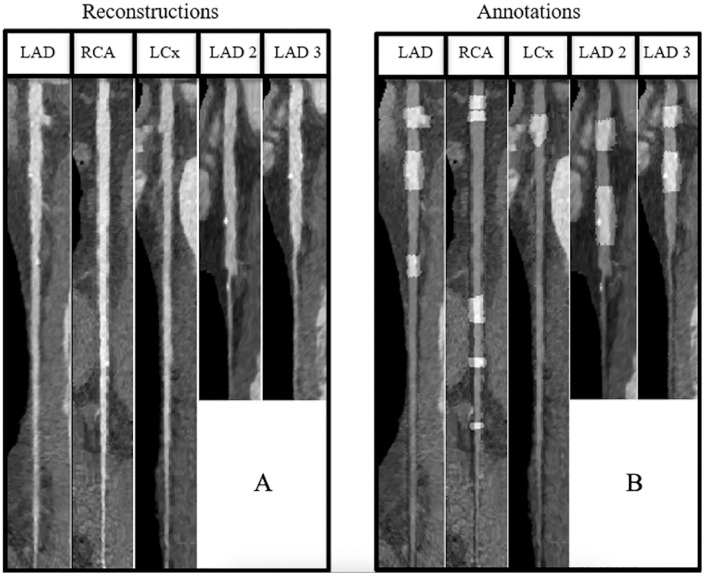
Example of stretched reconstruction of coronary arteries. 2D view of 5 largest arteries of a patient **(a)**, Annotation overlay on same arteries with light gray as segment with plaques and dark gray as non-pathological segments **(b)**.

Patients’ examinations have been fully anonymized and exported using commercially available software (Syngo.via, Siemens Healthineers, Erlangen, Germany) without any traceability. Annotations ranged from 0–2, with 0 for background, 1 for non-pathological segments of coronary arteries, and 2 for plaques. The database was divided into a negative database without any plaques and a positive database with the existence of at least one plaque (Figs 1 and 2).

### Data splitting

Due to the nnU-Net framework’s requirement for annotations for all training cases, only scans containing coronary plaques were included in the training set. To enhance the evaluation of negative predictability, 1613 individual coronary arteries from negative cases were added to the test dataset. The positive database was divided into training and test datasets, with an 80% and 20% split (using a fixed seed 42) based on patients’ IDs to prevent data leakage. The training dataset comprised axial 3D reconstructions of 4711 individual coronary arteries, while the test dataset included 1112 individual coronary arteries.

### Model training and hyperparameter tuning

The experiments were conducted on a Linux system (Ubuntu 18.04) equipped with 32GB RAM, an 8-core CPU (Intel® Core™ i7-9700K @ 3.60GHz), and an RTX 2080ti GPU. We used nnU-Net with Python 3.11 and PyTorch 2.1 with CUDA 11.8 versions matching nnU-Net requirements. Data preprocessing, integrity checks, and resampling to 1 mm^3^ isotropic spacing used nnU-Net’s built-in pipeline with CT-specific normalization.

The “3d_fullres” neural network architecture was used, implementing a PlainConvUNet model with a batch size of 20 and input patches of size 48x48x48. The network featured an encoder-decoder architecture with four stages, each containing two convolutional layers. It employed three max-pooling layers per axis during down sampling, with kernel sizes ranging from 1x1x1 to 2x2x2. Convolutional layers in both encoder and decoder stages used 3x3x3 kernels, with a maximum of 320 features. Data resampling and normalization were performed using nnU-Net’s CT-specific preprocessing pipeline, which resamples images to the target isotropic spacing using spline interpolation and applies global intensity normalization with clipping and z-score normalization [19]. During training, image augmentation was applied using the default nnU-Net data augmentation pipeline, which includes standard spatial and intensity-based transformations [19]. These settings follow the nnU-Net design, which selects patch sizes and network depth to balance anatomical context coverage and GPU memory constraints for a given dataset [19].

### Hyperparameter tuning and training

A 10% random subset of the training data (one-fold) was used for tuning (Table 1). The Dice similarity coefficient (1) was used as the standard overlap metric to evaluate the model’s performance after each parameter change. Hyperparameter tuning was restricted to parameters exposed by the nnU-Net framework, while core optimization settings such as optimizer type, initial learning rate, and weight decay were retained at their default nnU-Net values.

**Table 1:** Hyper parameter tuning.

Hyper parameters/	Default values for nnU-Net	Changes	Changes in validation Dice score
Batch size	47	6, 8, 12, 16, 20, 32, 64	0.78–0.80
Batch dice	Disabled	Enabled	0.80–0.80
Learning rate schedules	Linear	Cosine annealing	0.79–0.81
Epochs	1000	500, 1000, 1500	0.74–0.80
Optimizer	Stochastic Gradient Descent (SGD)	–	–
Weight Decay	3e-5	–	–
Initial learning rate	1e-2	–	–

We retained the default optimizer (SGD), initial LR (1e-2), and weight decay (3e-5). Cosine annealing and batch size of 20 yielded the highest mean Dice. Epochs exceeding 1000 did not improve the dice score.


$$\text{Dice}=\frac{\text{2}(TP)}{\text{2}(TP)+FP+FN}$$ (1)

### Training

The final model configuration used a batch size of 20 and a cosine annealing learning rate scheduler. We trained the nnU-Net model using a standard 5-fold cross-validation for 1000 epochs per fold.

### Cross-fold evaluation

To assess the stability of model performance across validation splits, we conducted non-parametric cross-fold comparisons using the Kruskal–Wallis test for all major metrics (Dice, Precision, Sensitivity, Specificity). The Kruskal–Wallis statistic quantified rank-based differences between validation sets and was interpreted in conjunction with the corresponding p-value [20]. All reported p-values exceeded the conventional significance threshold (α = 0.05), indicating no statistically significant differences in model performance across validation folds (Table 2).

**Table 2:** Cross-fold performance across validation splits.

Metric	Global Standard deviation	Global mean with 95% CI	Kruskal-Wallis Statistic (H)	P value
**Dice**	0.1529	0.75 (0.74, 0.75)	2.5578	0.6343
**Precision**	0.1630	0.77 (0.76, 0.77)	5.0482	0.2824
**Sensitivity**	0.1612	0.76 (0.76, 0.76)	1.9260	0.7494
**Specificity**	0.0026	0.99 (0.99, 0.99)	3.8666	0.4244

Global variability, confidence intervals, and Kruskal-Wallis comparison.

### Inference and evaluation

For inference, nnU-Net used an ensemble of all five cross-validation models, with predictions averaged across folds [19]. Post-processing applied binary opening, a morphological operation consisting of erosion followed by dilation, using a 3 × 3 × 3 structuring element for three iterations to remove small spurious predictions. An ablation analysis, in which the number of binary opening iterations was varied from 1 to 6 on a validation set generated during training, confirmed that three iterations provided a balance between false positive suppression and preservation of plaque integrity.

### Evaluation methodology

Given the anatomical complexities of coronary arteries, which span across the middle of a 3D matrix, a 3D radius dilation mask centered on vessel centerlines was introduced. This matrix aimed to preserve the center-lined coronary arteries while eliminating noise or partial annotations from neighboring vessels. Leveraging the Labeling function within the SciPy library, individual connected segmentations were separated and labeled within both ground truths and predictions.

## Results

### Plaque-level evaluation

**Fig 3:**
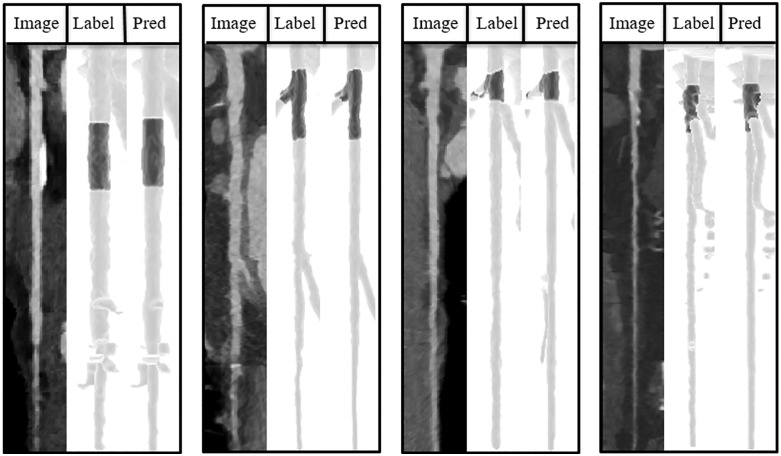
Examples of Predictions with dice scores more than 0.88. The model showed a promising performance in finding medium to large plaque annotations.

**Fig 4:**
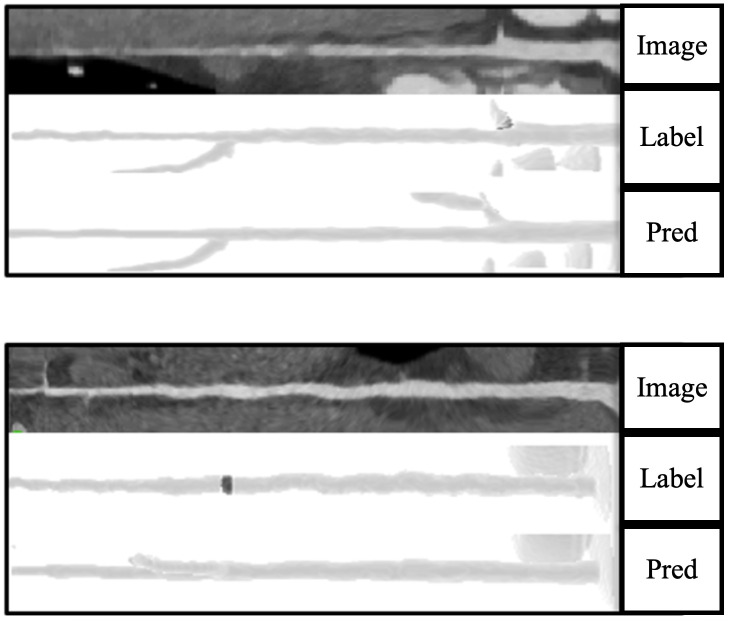
Example of small plaque annotations (< 99 mm^3^). The model has failed to identify some smaller non-calcified plaques.

From a total of 2090 plaques across 1112 vessels in our test dataset, the neural network model correctly identified 1772 plaques (TP) (Fig 3). It failed to identify 318 plaques (FN) and mistakenly marked 382 parts of coronary arteries as plaques (FP) that had no overlap with ground truth annotations (Fig 4).

### Vessel-level evaluation

Using a dataset of 1613 negative vessels, the model correctly identified 1369 as normal and misclassified 244 as pathological (specificity = 84.9% (95% CI 83.0–86.5)). Out of 1112 positive vessels, the model correctly marked 1053 as positive but failed to detect plaques in 59 positive vessels (sensitivity = 94.7% (95% CI 93.2–95.9%)). The model demonstrated a positive predictive value (PPV (2)) of 81.2% (95% CI 79.0–83.2%) at the vessel level.


$$PPV=\frac{TP}{TP+FP}$$ (2)

### Examination-level evaluation

Among 231 patients with coronary artery disease, the model successfully identified 225 as positive (at least one true positive vessel) but missed 6 positive patients. In the negative dataset, the model correctly classified 530 patients as negative but incorrectly marked 135 as having CAD. Patient-level analysis showed a standard deviation of 1.19 for FP distribution and 1.2 for FN distribution over examinations.

In negative patients, 79.7% had zero FP plaques, while 20.3% had one or more FP plaques, primarily one or two, indicating instances of over-detection. The model demonstrated an NPV (3) of 98.8% (95% CI 97.6–99.5%) at the examination level.


$$NPV=\frac{TN}{TN+FN}$$ (3)

### Segmentation performance and error analysis

**Fig 5:**
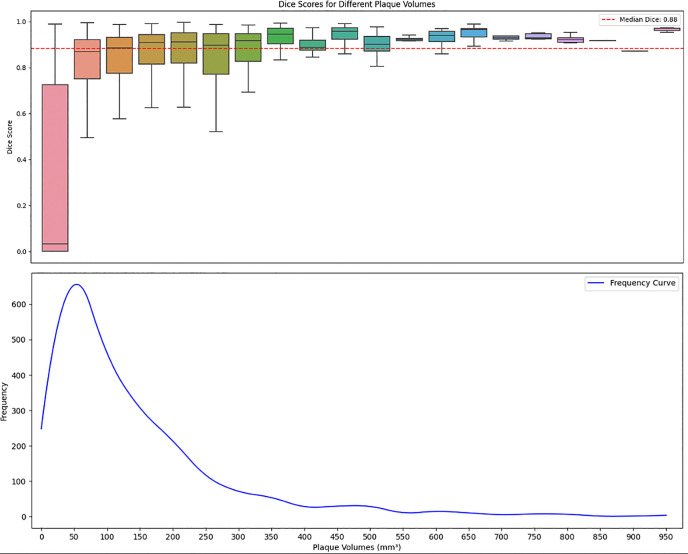
Illustration of model’s performance. Dice score distribution based on plaque annotation volumes (top). A frequency curve illustrated the distribution of plaque volumes within the dataset (bottom).

We evaluated the model’s plaque segmentation performance using Dice score distribution for segmentation size and presence of calcifications. This Analysis showed coherent trends across subgroups. Non-calcified plaques (0 mm^3^) had a higher false-negative rate of 39.4% with a median Dice of 0.66 (95% CI 0.59–0.74), whereas segmentations containing larger than 5 mm^3^ calcification demonstrated a 0% false-negative rate and a Dice of 0.93 (95% CI 0.92–0.93) (Table 3). Size-based assessment indicated that small plaques segmentations (<200 mm^3^) accounted for 97.16% of false negatives, while segmentations larger than approximately 400 mm^3^ did not exhibit false-negative errors in our dataset (Table 4). The model achieved a median Dice score of 0.88 (95% CI 0.87–0.89) across all plaque volumes (Fig 5).

**Table 3:** Detection performance stratified by calcification volume.

Calcification volumes (mm³)	Counts	Number of TP plaques	Number of FN plaques	FN rate %	Median Calcification with 95% CI	Median Dice with 95% CI
**0**	558	337	221	39.60	0.00 (0.00, 0.00)	0.66 (0.59, 0.74)
**>0–5**	996	899	97	9.73	1.25 (1.25, 1.37)	0.88 (0.87, 0.89)
**>5**	536	536	0	0.00	11.43 (10.12, 12.25)	0.93 (0.92, 0.93)

Metrics are computed per plaque; confidence intervals use bootstrap estimates.

**Table 4:** Detection performance stratified by plaque volume.

Volume Interval (mm³)	Counts	Number of TP plaques	Number of FN plaques	FN rate %	Median Calcification (mm³) with 95% CI	Median Dice with 95% CI
**0–200**	1515	1206	309	20.39	0.50 (0.37, 0.62)	0.89 (0.88, 0.90)
**200–400**	432	423	9	2.08	6.25 (5.37, 7.31)	0.91 (0.91, 0.93)
**400–600**	96	96	0	0.00	22.87 (20.37, 28.75)	0.91 (0.90, 0.93)
**600–800**	36	36	0	0.00	43.25 (35.93, 52.12)	0.93 (0.92, 0.95)
**800–1000**	11	11	0	0.00	69.50 (27.75, 101.25)	0.92 (0.89, 0.94)

All values are plaque-level; confidence intervals use bootstrap estimates.

## Discussion

In this study, we developed and evaluated a neural network model based on nnU-Net for automated detection and segmentation of coronary plaques in CCTA images.

Our results compare favorably with previously reported performance in the literature on automated plaque detection and segmentation. Liu et al. reported a 3D convolutional network that categorized coronary plaques as calcified, mixed, or non-calcified, achieving Dice scores per vessel of 0.83, 0.68, and 0.73, respectively [21]. In comparison, our model achieved higher Dice scores, particularly for larger plaques, indicating improved segmentation accuracy. Masuda et al. demonstrated a convolutional neural network with an accuracy of 0.86 and an F1 score of 0.85 in plaque detection [22]. For binary segmentation tasks, the Dice similarity coefficient is mathematically equivalent to the F1-score, allowing direct comparison with these results. Zreik et al. reported an accuracy of 0.80 for detecting coronary stenosis [23]. Our model showed comparable performance in these aspects.

Dey et al. [14] used machine learning (ML) to predict lesion-specific ischemia, integrating various quantitative plaque measures from coronary CTA. Their model achieved an area-under-the-curve (AUC) of 0.84, outperforming individual CTA measures. While our study did not directly assess ischemia prediction, our accuracy in plaque detection and segmentation suggests that our model could potentially serve as a valuable input for such ischemia prediction models, potentially improving their performance further.

Hong et al. [24] developed a deep learning approach for stenosis quantification from coronary CTA, achieving excellent correlations with expert readers for minimal luminal area, percent diameter stenosis, and percent contrast density difference. While their focus was on stenosis quantification, our model’s strength lies in comprehensive plaque detection and segmentation. The high performance of both approaches suggests that deep learning methods can effectively automate various aspects of CCTA analysis, potentially complementing each other in comprehensive CAD assessment.

The high negative predictive value of our model suggests its potential utility as a cost-effective exclusion test for CAD. This aligns with the findings of van Velzen et al. [25], who reported high performance in assigning cardiovascular disease risk categories from cardiac CT, although they investigated calcium score CT only. Our model’s ability to accurately identify patients without plaques and thus without coronary artery disease could be particularly valuable in clinical settings for rapidly triaging patients and potentially reducing the need for unnecessary invasive procedures.

## Limitations

Several limitations merit discussion.

First, the detection of smaller non-calcified plaques remains challenging, as evidenced by a higher occurrence of false negatives in this subgroup. Such plaques represent early atherosclerotic changes. The subtle intensity differences and variable arterial geometry complicate accurate segmentation. Addressing this challenge will require enriched training datasets emphasizing small plaque examples, as well as advanced loss functions or attention mechanisms to prioritize detection of smaller non-calcified components. For example, focal loss has been shown to improve sensitivity for under-represented targets by down-weighting easy background examples [26], while attention mechanisms can help networks focus on subtle, localized features [27]. Although such approaches have shown promise in prior work, they are not part of the standard nnU-Net framework, which relies on Dice and cross-entropy loss.

**Fig 6:**
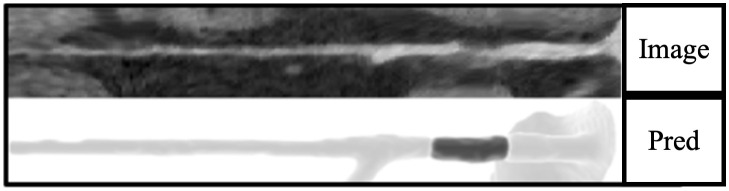
False positive prediction. A motion artifact which caused a false prediction as plaque.

Second, image quality factors—including motion or step artifacts and severe vessel narrowing at branch points—contributed to false positives. We found that approximately 30% of false positives did not correspond to actual pathology; the remaining false positives were due to artifacts such as step or motion artifacts (Fig 6). Although experienced radiologists can correctly identify most artifactual detections as non-pathological (e.g., step artifacts), some coronary segments may remain non-diagnostic due to limited image quality (e.g., blooming from calcifications or motion-related artifacts). In such cases, further evaluation using alternative methods, such as invasive coronary angiography or stress imaging, may be warranted. Third, the model was trained and evaluated using data from a single center. No publicly available annotated CCTA dataset for coronary plaques was accessible at the time of the study. To support independent assessment, we provided a sample dataset and pipelines for database conversion and inference, which enable other centers to prepare their internal data for model testing.

Furthermore, our reliance on stretched (straightened) reconstructions—though standard on many vendor platforms—limits direct applicability to native curved MPR images. While stretched reconstructions may not be the primary viewing method, they serve as an additional analytical tool that complements, rather than replaces, traditional visualization [28,29]. Our method can be seamlessly integrated into existing workflows, with results mapped back to conventional views by reversing the mathematical transformation. We plan to extend our pipeline into a fully automatic segmentation tool that operates directly on the original MPR data, extracts individual coronary arteries, applies segmentation, and subsequently maps all identified plaques back onto the original MPR images.

## Conclusion

Our nnU-Net-based model demonstrates strong segmentation performance for medium-to-large and calcified coronary plaques in CCTA, with high negative predictive value for ruling out significant disease. Performance remains limited for small and non-calcified lesions, and variability in image quality reduces reliability in challenging cases. Considering this limitation, translation toward clinical use will require further work, including improvements in detecting these lesion types through training and validation on multi-center datasets and systematic comparison against expert radiologists. We believe that the presented results could help encourage multi-center collaborations and support future progress toward semi-automated plaque detection in coronary CTA.
